# Magnets in action: role of interventional radiologists in magnetic compression anastomosis procedures

**DOI:** 10.1186/s13244-024-01705-9

**Published:** 2024-05-31

**Authors:** Emre Ünal, Türkmen Turan Çiftçi, Devrim Akinci, Erkan Parlak

**Affiliations:** 1 Department of Radiology, Faculty of Medicine, Hacettepe University, Ankara, Turkey; 2 Department of Gastroenterology, Faculty of Medicine, Hacettepe University, Ankara, Turkey

**Keywords:** Magnetic compression anastomosis, Impassable obstruction, MCA, Magnamosis, Magnet

## Abstract

**Abstract:**

Obstructions encountered in biliary, gastrointestinal, and urinary tracts are increasing in number due to successful percutaneous and endoscopic organ-saving procedures. Although functional recovery is established to an extent, failure of traversing an obstruction may end up necessitating invasive surgical procedures. Multidisciplinary collaboration may traverse the limitations of each individual approach, therefore creating the perfect intervention for the patient. Magnetic compression anastomosis is a minimally invasive procedure that can provide a great outcome in select cases with biliary, gastrointestinal, or urinary tract obstructions.

**Critical relevance statement:**

In this article, various applications of magnetic compression anastomosis are reviewed with illustrative cases of esophageal, biliary, colonic, and urinary obstructions that cannot be traversed with a wire. This method will expand the spectrum of interventions performed in the IR unit.

**Key Points:**

- Magnets can enable wire access beyond an impassable obstruction.
- Magnets can create anatomical and non-anatomical anastomosis at an occlusion.
- Magnetic compression anastomosis is a minimally invasive procedure that can provide great outcomes.

**Graphical Abstract:**

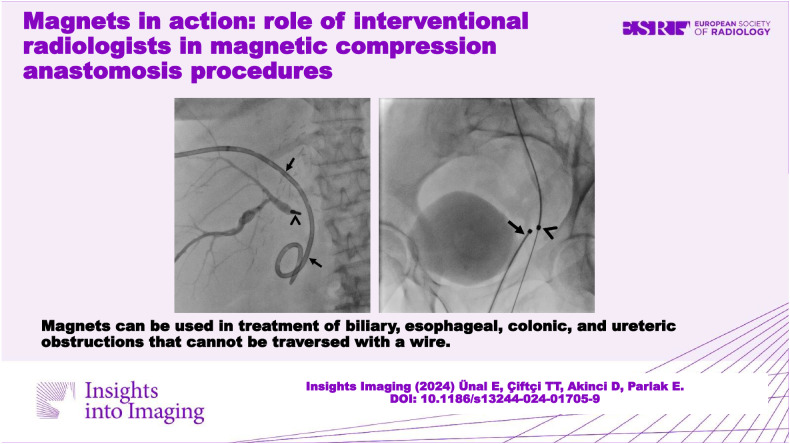

## Introduction

Magnetic compression anastomosis (MCA, Magnamosis) is performed as a two-step procedure. First, two rare-earth magnets are placed at the cranial and caudal parts of an obstruction. In this state magnets face each other with their magnetic attraction capacity. In the second procedure, the occlusion is traversed by manipulating a guiding catheter over the guidewire. The second step of the procedure is scheduled when the magnets demonstrate adherence without intervening tissue on a plain radiograph. Therefore, plain radiographs are routinely obtained to evaluate the magnets’ position. In the literature, MCA has gained attention and popularity due to its minimally invasive nature [1]. One critical issue is the distance of the stricture, which should not be beyond the magnetic attraction capacity of the magnets; therefore, patients with malignant tissue infiltrating obstructions may not be perfect candidates for MCA [2–4].

MCA is currently used in the treatment of biliary, esophageal, colonic, and ureteric obstructions [1–12]. In addition, MCA has also been used for diversion procedures such as gastrojejunostomy or jejunojejunostomy [6, 13, 14].

Magnets can create anatomical and non-anatomical anastomosis at an occlusion. Animal studies have revealed that full-thickness anastomosis with serosal apposition can be achieved with MCA [15–17]. In addition, biopsy specimens obtained from the site of MCA demonstrated rapid epithelization and decreased inflammation which indicates longer patency expectancy [7, 18]. Although published data point out the efficacy of the MCA procedure, there is still a lack of data regarding long-term follow-up findings of the reconstructed anastomosis. Nevertheless, Jang et al reported a lower stricture recurrence rate after MCA compared to conventional methods [1].

In this article, various applications for MCA are reviewed with illustrative cases.

### Hepatobiliary obstruction

The majority of literature regarding MCA primarily focuses on biliary obstructions. Liver transplant recipients, in particular, suffer from biliary complications and impassable obstructions are not uncommon [19, 20]. In addition, iatrogenic injury of the biliary system during cholecystectomy may result in significant biliary stenosis and complete biliary obstruction in case of clipped bile duct [21]. MCA can be applied to both native and transplanted livers, however, rigorous assessment of the obstruction is essential for technical success. Endoscopy plays a more crucial role in the MCA of hepatobiliary obstructions compared to esophageal and ureteric obstructions. Retrograde catheterization of the cystic duct and afferent loop are essential steps of the procedure in the case of the clipped aberrant bile duct and Roux-en-Y hepaticojejunostomy, respectively.

### Native liver

**Fig. 1:**
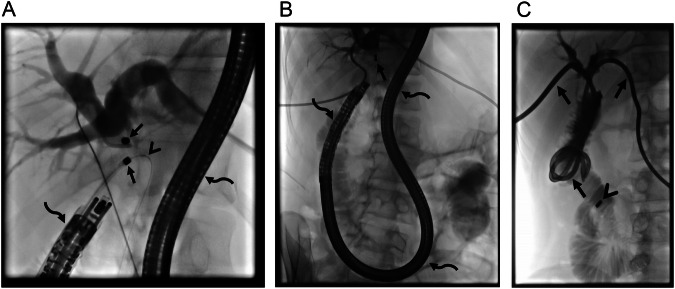
A 25-year-old man developed hepaticojejunostomy anastomosis obstruction 6 months following surgery for type 1 choledochal cyst and pancreaticobiliary maljunction. Initially, biliary drainage was performed, however, the obstruction could not be traversed despite multiple manipulations of various guidewires. MCA was considered for treatment. **A**, **B** An adult colonoscope with a 3.8 mm working channel was successfully placed in the Roux limb (curved arrows, **A**, **B**) and a guidewire (arrowhead, **A**) was able to be advanced to the caudal part of the obstruction. Magnets (10 F) were successfully placed under fluoroscopy (arrows, **A**, **B**). **C** On the 6th day, the obstruction was able to be traversed and bilateral biliary drainage catheters were placed (arrows). Note spontaneously dropped magnets in the lumen of the small bowel (arrowhead)

**Fig. 2:**
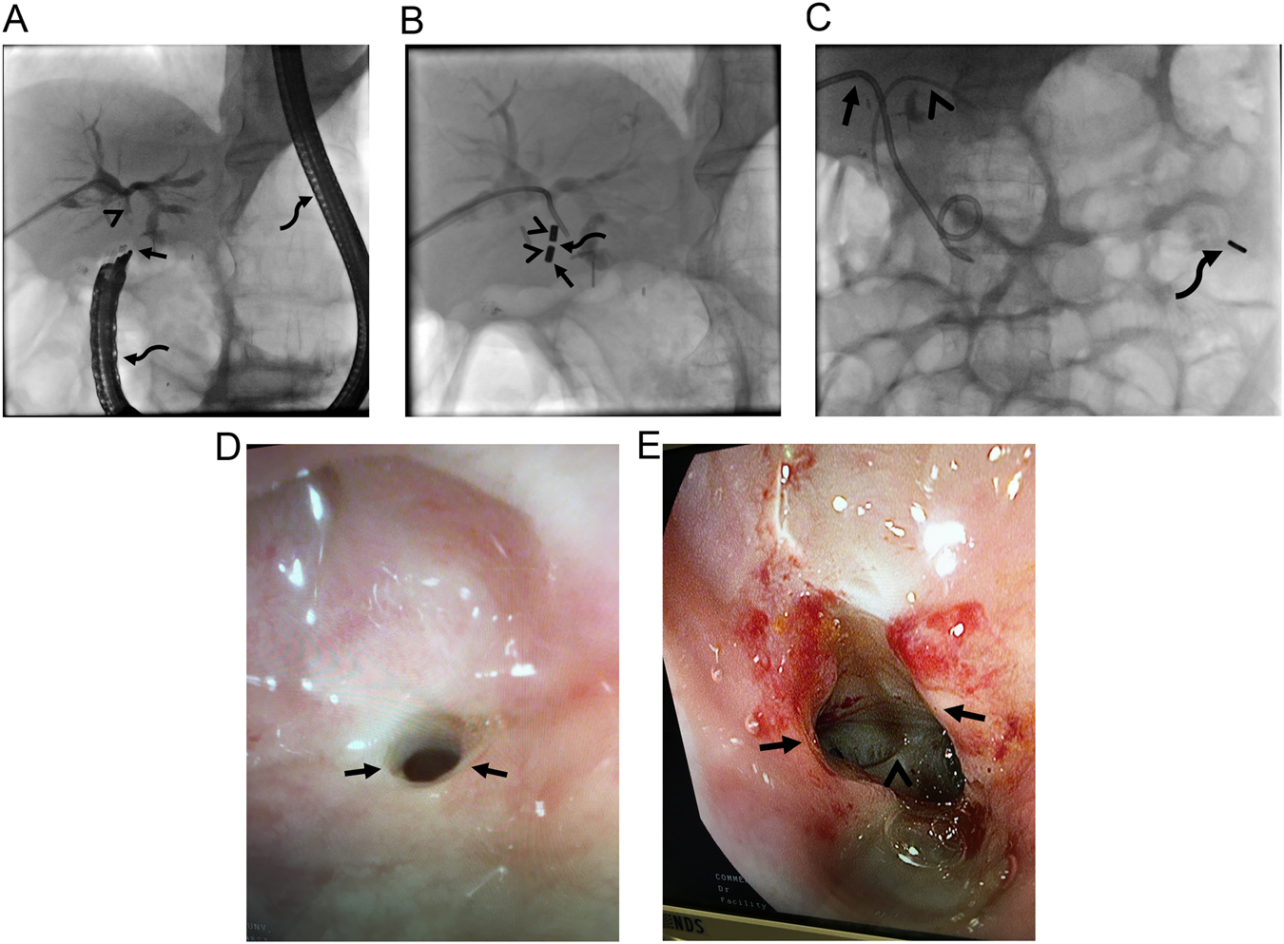
A 68-year-old man underwent a Whipple procedure for pancreatic cancer. He developed hepaticojejunostomy anastomosis obstruction 8 months following surgery. **A**, **B** Percutaneous biliary access failed to traverse the bilioenteric anastomosis obstruction (arrowheads, **A**). However, endoscopic access and consequently magnet placement at the caudal part of obstruction through colonoscope was able to be achieved (arrows, **A**, **B**). Magnets (7 F) demonstrated adequate attraction to each other (arrowheads, **B**). Note intervening soft tissue between magnets (curved arrow, **B**). **C** On the 7th day magnets demonstrated adherence (not shown) and right-sided 10 F internal biliary drainage catheter (arrows) and left-sided 7 F plastic stent (arrowheads) were able to be placed. Note spontaneously dropped magnets in the lumen of bowel segments (curved arrow). **D**, **E** Anastomosis demonstrated significant enlargement on follow-up (arrows, **D**, **E**). Note intrahepatic bile ducts on the endoscopic image (arrowhead, **E**). An endoscopic biopsy performed in 6th month revealed epithelialization and lack of inflammation at the anastomosis (not shown). The patient has been symptom and stent-free for almost 3 years

MCA in the management of biliary obstruction encountered in native livers is relatively rare compared to transplanted livers. Post-surgical bilioenteric anastomosis strictures and traumatic injuries account for the majority of cases. Percutaneous biliary interventions are the initially preferred treatment options for bilioenteric anastomosis strictures because endoscopic retrograde cholangiopancreatography (ERCP) is challenging in this patient population due to altered anatomy, like in Roux-en-Y hepaticojejunostomy. In the presence of impassable biliary obstructions in these patients, MCA is challenging due to the limitations of ERCP. However, using a long endoscope (colonoscope or enteroscope) may enable access to the stricture site through the afferent loop, even in Roux-en-Y reconstructions [22, 23]. Although ERCP access may not be adequate for stent insertion or further interventions, MCA can still be achieved if a guidewire to carry one of the magnets can be advanced to the stricture site with the access obtained with the colonoscope (Figs. 1 and 2).

**Fig. 3:**
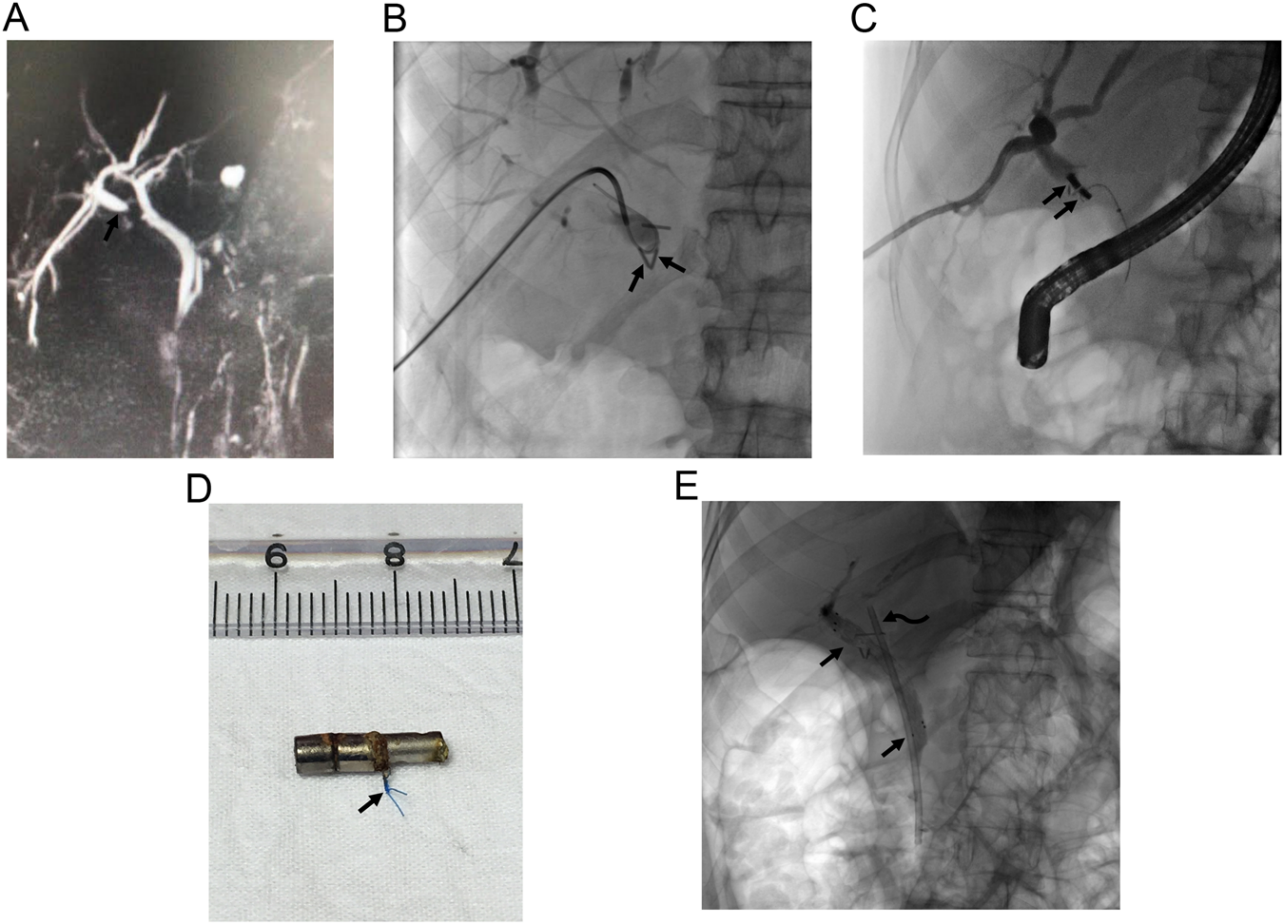
Strasberg type B injury in a 57-year-old woman. She presented with acute cholangitis 4 months following laparoscopic cholecystectomy surgery. **A** MRCP revealed isolated right posterior bile duct dilatation (arrow). **B** Bile duct dilation and clinical signs related to acute cholangitis relieved following percutaneous biliary drainage, however, both antegrade and retrograde access failed in traversing the clipped bile duct (arrows). **C** Magnets were successfully placed (arrows). The caudal magnet (5 F) was smaller in size compared to the cranial magnet (7 F) due small caliber of the cystic duct. **D** A suture thread (arrow) found between magnets indicates anatomic anastomosis with MCA. **E** The patient was able to be discharged without an external drain. Note fully covered self-expandable metallic stent (arrows) lying between the right posterior bile duct and the main bile duct. The curved arrow indicates a left-sided plastic stent

**Fig. 4:**
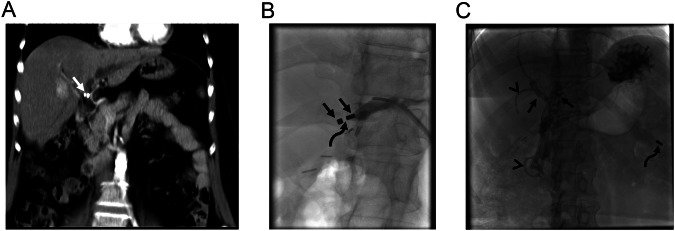
Strasberg type E4 injury in a 60-year-old woman. The iatrogenic injury also resulted in right hepatic artery occlusion (not shown). **A** The left hepatic artery was patent however, the left main bile duct was clipped (arrows). ERCP was successful in placing two plastic stents in the right lobe bile ducts (not shown), however, failed in catheterization of the left lobe bile duct. **B** MCA was successfully performed (arrows). Note intervening soft tissue between magnets (curved arrow). **C** Two fully covered self-expandable metallic stents (arrows) and a rescue double pigtail plastic stent (arrowheads) were successfully placed following MCA. Note magnets in the lumen of proximal jejunal segments (curved arrow)

Bile duct injury during cholecystectomy may also result in complete biliary obstruction due to clipped bile duct, particularly in patients with biliary anatomical variations [21]. Several types of biliary duct variations have been identified and right accessory biliary duct variations are held responsible for the majority of cases of bile duct injury during laparoscopic cholecystectomy [21, 24]. Ligation or clipping of aberrant right biliary truncus during cystic duct ligation in these patients results in isolated accessory biliary obstruction due to hampered bile drainage from these ducts to the main bile duct. MCA between the remaining part of the right accessory biliary truncus and the cystic duct may establish restored bile flow (Fig. 3). In addition, direct iatrogenic injury to the common bile duct may result in complete obstruction if left untreated (Fig. 4). The treatment commonly ends up with hepaticojejunostomy if the injury is detected during surgery. However, it may be diagnosed in the post-surgical period, and in this setting, rapid intervention should be performed because injury may result in retraction of the bile ducts which can preclude MCA.

### Transplant liver

**Fig. 5:**
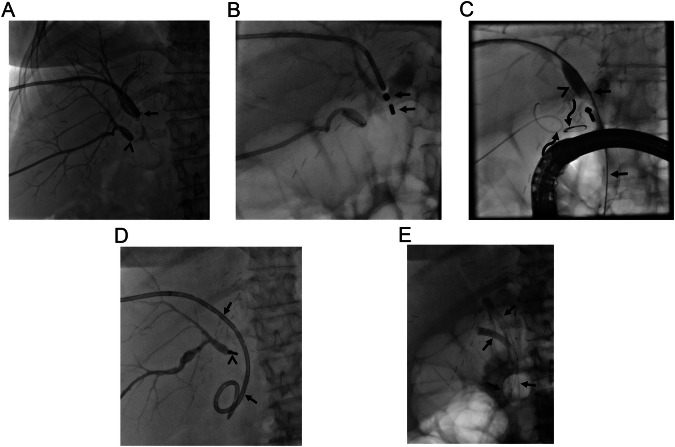
A 59-year-old woman underwent living donor liver transplantation surgery 10 months ago, and presented with pruritus and cholangitis. Liver MRI (not shown) demonstrated biliary obstruction. **A** Initially performed percutaneous biliary drainage revealed obstruction of two duct-to-duct anastomoses at the recipient’s main bile duct (arrow) and cystic duct (arrowhead). Both antegrade and retrograde attempts failed in traversing the obstruction. **B** First, magnets are placed at the anastomosis between the transplanted liver and the recipient’s main bile duct (arrows). **C**–**E** On the 5th day MCA was formed and antegrade wire access through the main bile duct was achieved (arrows, **C**). A balloon catheter (arrowhead, **C**) was used to push down the magnets. Consequently, a biliary drainage catheter was able to be placed (arrows, **D**). The second procedure was planned for MCA for cystic duct anastomosis obstruction (curved arrows, **C**). On the 6th day following magnet placement at cystic duct anastomosis (arrowhead, **D**), two fully covered self-expandable metallic biliary stents were able to be placed (arrows, **E**) and percutaneous drains were removed

Several risk factors are held responsible for biliary complications following transplant surgery. Small and multiple anastomoses in patients with living donor transplantation, post-surgical adhesions/inflammation may result in biliary anastomosis stricture, and hepatic artery-related complications (i.e., decreased arterial blood supply or occlusion) may worsen the degree of stricture. In addition, the presence of a bile leak may result in biliary anastomotic stricture which may rapidly evolve to complete obstruction. Biliary strictures following liver transplantation can be encountered as anastomotic or non-anastomotic strictures. Anastomotic strictures are more common and more manageable with percutaneous and endoscopic procedures. Non-anastomotic strictures may occur due to various etiologies and have poorer prognosis. Management may vary depending on the type of anastomosis which can be performed as duct to duct [common biliary duct (CBD) of the donor to CBD of recipient] or hepatico/choledecho-jejunostomy or anastomosis of donor CBD to recipient’s cystic duct [4]. Most biliary anastomotic strictures are managed with endoscopic procedures. Percutaneous biliary interventions are reserved for failed ERCP cases or patients with hepaticojejunostomy anastomosis. Impassable complete biliary obstructions are not uncommon in liver transplant recipients. MCA may prevent risks of revision surgery, which is the conventional treatment option in these patients (Fig. 5).

### Urinary obstruction

Recently published literature has revealed the success of MCA in ureteric obstructions [2, 12, 25]. Although the majority of ureteral obstructions can be traversed with guidewires, impassable strictures can be encountered. In contrast to biliary occlusions, endoscopic guidance is generally not required in the management of ureteral obstructions with MCA. With the latest technical improvements, the distal ureter can be catheterized under imaging guidance with only a few steps using several elemental tools [26]. One issue that should be addressed regarding the MCA of urinary obstruction is infection related to long-standing nephrostomy catheters. It may be encountered in hepatobiliary obstructions to some extent, however, it is not expected to be encountered in MCA of gastrointestinal obstruction due to a lack of drainage catheters.

### Native ureter

**Fig. 6:**
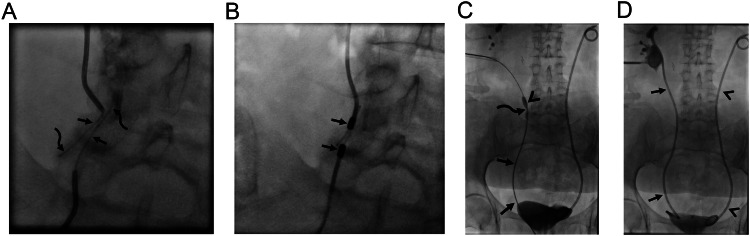
A 58-year-old woman underwent surgery and received chemoradiotherapy for endometrial carcinoma. She developed bilateral hydroureteronephrosis on follow-up. **A** An 8 F double J stent was successfully placed in the left ureter however, both antegrade and retrograde accesses failed in traversing right ureteral obstruction (arrows, **A**), therefore patient opted for percutaneous nephrostomy (not shown). False lumen beyond the obstruction is also noted (curved arrows, **A**). **B** Magnets were placed at the site of obstruction (arrows). **C** Compression anastomosis was successfully formed and the obstruction was able to be traversed (arrows). Afterward, balloon dilatation was performed prior to double J stent placement (arrowheads). Note the waist during balloon dilation (curved arrow). **D** An 8 F double J stent was able to be placed (arrows). Left-sided double J stent is also noted (arrowheads)

**Fig. 7:**
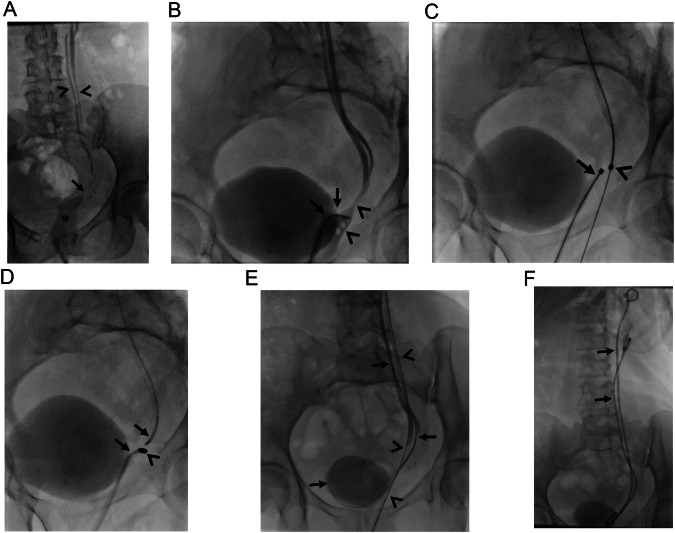
A 49-year-old woman with a history of cervical carcinoma. **A** Chemoradiotherapy resulted in a complete response of pelvic tumor, however, UVF was encountered on follow-up (arrows). The asterisk indicates the vagina. The patient had also incomplete ureteral duplication (arrowheads). Ureter orifices could not be catheterized with cystoscopy. Reconstruction surgery was avoided considering the comorbidity. MCA was planned. **B** The caudal part of the ureter was short (arrows) and demonstrated retraction as a consequence of chronic UVF (arrowheads). **C** A stiff straight tip wire was introduced through the ureteral trigon (arrow) and the magnet was held in position until the cranial magnet was in place (arrowhead). **D** Consequently, both wires were pulled back while magnets were held in position with the support of guiding catheters (arrows). Magnets demonstrated rapid adherence without intervening soft tissue (arrowhead). Magnets fell spontaneously on the 3rd day. **E**, **F** Both antegrade (arrows, **E**) and retrograde (arrowheads, **E**) accesses were used for double J stent placement for each ureter (arrows, **F**)

Radiotherapy in the treatment of gynecological malignancies may result in significantly narrowed ureters [27, 28]. Care should be taken during the catheterization of ureteral strictures because ureteral integrity can be disrupted during guidewire insertion and the procedure may end up creating a false lumen and associated total ureteral occlusion. Once the false lumen is formed, it may be challenging to find the true lumen beyond the stricture (Fig. 6). Ureterovaginal fistula (UVF) is also another unfortunate complication encountered during the follow-up of patients who have undergone gynecological surgery and/or radiotherapy [27–31]. Surgical treatment is usually avoided preferring less invasive treatment options such as imaging or cystoscopically guided procedures [29–31]. Time of diagnosis is crucial in the treatment of ureterovaginal fistulae. Once a fistula is identified, interventions should be carried out as soon as possible to preclude fistula maturation [29–31]. In the chronic state, any wire access beyond the fistula will probably fail to establish urinary diversion. However, MCA may restore the connection between the bladder trigone and the caudal part of the ureter even in chronic UVFs (Fig. 7).

### Post-surgical urinary anastomosis

**Fig. 8:**
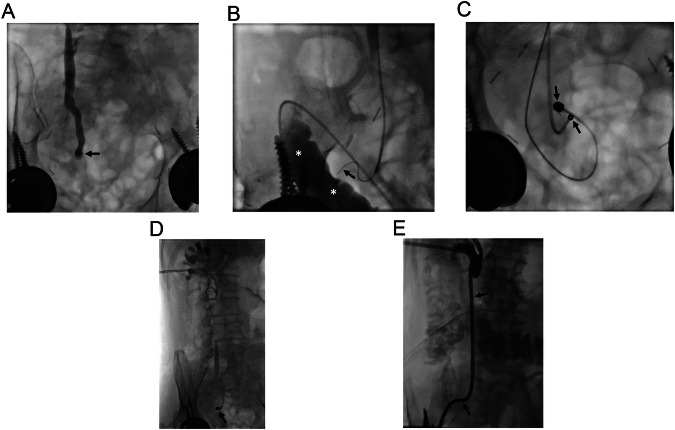
A 76-year-old man developed right hydroureteronephrosis 10 months following cystoprostatectomy with ileal conduit surgery. **A**, **B** MCA was planned due to the failure of traversing ileoureteral anastomosis obstruction (arrows, **A** and **B**). Asterisks indicate an ileal conduit. **C**–**E** Magnets (arrows, **C**) demonstrated adherence on the 5th day (arrow, **D**), and consequently, retrograde 10 F nephroureteral stent (arrows, **E**) was able to be placed

Cystectomy with ileal conduit is a type of surgery performed for patients with bladder cancer. The ureters are anastomosed to a reconstructed bladder which is placed at lower abdominal quadrants. The presence of inflammation and/or urinary leak, surgical challenges, and errors are held responsible for ileoureteral anastomosis strictures. These strictures can be traversed with antegrade or retrograde access gained with a nephrostomy or existing ostomy, respectively [32, 33]. During catheterization of the ileoureteral anastomosis stricture, care should be given to prevent extravasation which may result in distraction of the ureter from the anastomosis. In this case, patients may end up with permanent nephrostomy. Surgery is generally avoided in ileoureteral anastomotic obstructions, and interventional radiologists play a crucial role in the management of these obstructions [2, 32, 33]. Ileal pouch with an existing ostomy enables insertion of larger-sized magnets compared to native ureters, and therefore, more severe strictures may be treated with MCA due to enhanced attraction capacity with larger magnets (Fig. 8). MCA has also been reported as an effective procedure in renal transplant recipients with total ureteral obstructions [12].

### Gastrointestinal system obstruction

**Fig. 9:**
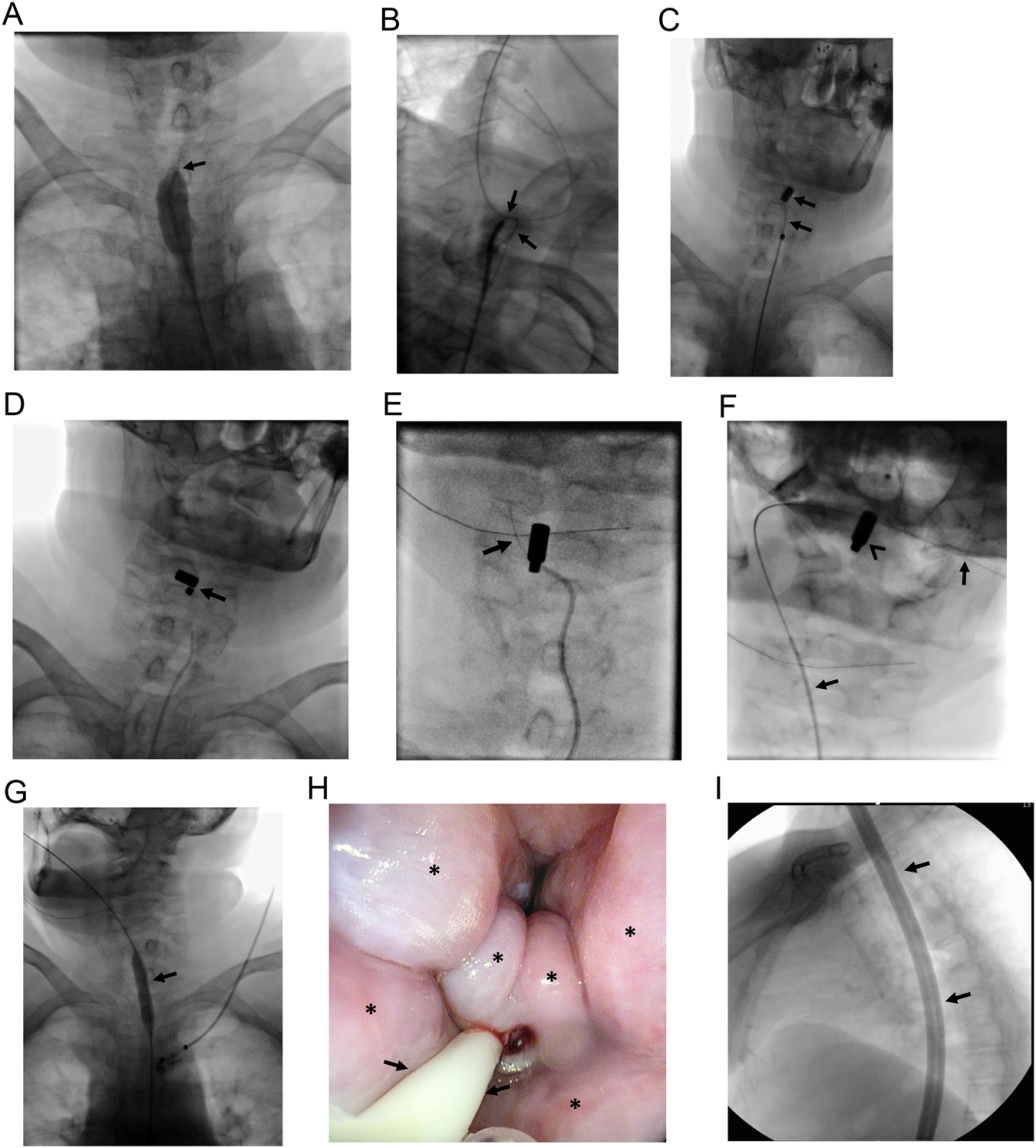
An 85-year-old woman with a previous history of surgery and radiotherapy for hypopharynx cancer was referred for spitting out oral secretions. A gastrostomy tube was placed two years ago for nutrition. Initial endoscopy revealed hypertrophic mucosa and a lack of luminal continuity through the esophageal lumen (not shown). **A** Retrograde access through gastrostomy was also unsuccessful in traversing the obstruction (arrow). MCA was decided. **B**–**D** Two magnets were delivered with the support of stiff guidewires, at the cranial and caudal parts of the occlusion under fluoroscopy guidance (arrows, **B**–**D**). **E**–**F** On the 6th day following magnet placement, occlusion was able to be traversed with a hydrophilic guidewire (arrow, **E**), and the magnet migrated (arrowhead, **F**) through the tongue by itself following through and through access (arrows, **F**). Afterward, the magnets were removed with digital manipulations. **G**, **H** Balloon dilatation was performed (arrow, **G**) a 10 F nasogastric tube was advanced to the stomach (arrows, **H**) and gastrostomy was replaced. Hypertrophic mucosa is seen on endoscopic images (asterisks, **H**). On the 2nd day following MCA, the patient was able to swallow oral secretion. **I** On the 10th day, bougie dilatation (arrows) was performed and both the nasogastric tube and gastrostomy were removed. The patient is still symptom and gastrostomy-free during 16 months of follow-up

Esophageal obstructions are encountered following treatment of head and neck cancers and esophageal atresia, in adult and pediatric patient populations, respectively. Esophageal strictures are frequently managed with bougie and balloon dilation or stenting if wire access through the stricture can be established. However, in case of failure, surgical resection and reconstruction may be required which is a complicated procedure considering patient performance status and surgical challenges. MCA in the management of gastrointestinal obstruction has been reported even in pediatric patient populations [7–9]. The procedure is similar to the above-mentioned obstructions. In patients with esophageal obstruction, a gastrostomy tube is inserted for enteral nutrition also enabling access to the caudal part of esophageal obstruction. The access to the cranial part of esophageal obstruction is more straightforward with the use of endoscopy or fluoroscopy-guided wire access. Various types of esophageal obstruction including esophageal atresia, post-surgical complication, or RT-induced obstruction can be treated with MCA (Fig. 9).

**Fig. 10:**
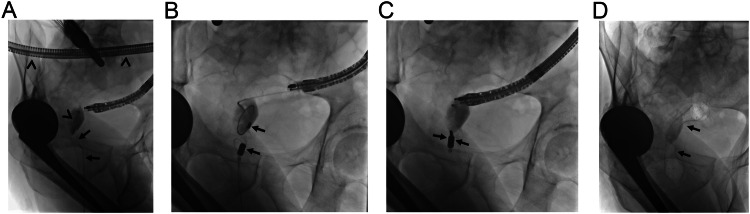
A 61-year-old man with a previous history of low anterior resection and double barrel ostomy surgery for rectal carcinoma. He received chemoradiotherapy. During pre-operative assessment for ostomy closure, obstruction was encountered at anastomosis (not shown). **A** Both imaging-guided retrograde access (arrows) and endoscopy advanced through the existing ostomy (arrowheads), failed to traverse the obstruction, therefore MCA was decided. **B** Two magnets were delivered at the cranial and caudal parts of the occlusion under fluoroscopy guidance (arrows). **C** The magnets adhered instantly without intervening tissue (arrows). On the 3rd day, magnets discharged through the anus spontaneously. **D** Consequently, the obstruction was able to be traversed and a self-expandable fully covered metallic colonic stent was placed (arrows). The colostomy closure procedure was uneventful. The patient found a colonic stent in his stool 10 days after the closure surgery, and even since he is symptom-free

MCA has been reported to be a safe and effective treatment option for small and large intestinal obstructions, as well [6, 13, 34]. Gastrojejunostomy can be reconstructed with MCA in patients with periampullary region tumors to enable oral nutrition [6, 13, 14]. Duodenal stent placement may not remain patent or overcome the obstruction in case of long-segment involvement. Therefore, these patients may undergo endoscopic ultrasound-guided gastrojejunostomy or surgical gastrojejunostomy which has its own disadvantages and challenges. However, several studies have demonstrated that MCA can be successfully performed to create an anastomosis between the stomach and jejunum, two different jejunal segments, or colonic segments in a minimally invasive manner [5, 6, 13, 34, 35]. However, the placement of a metallic stent following MCA in small intestinal obstructions remains controversial [6, 13, 14]. It should be noted that premature dilatation of MCA between gastrointestinal segments may result in perforation, which may end up with severe comorbidity and even death. Therefore, in the setting of gastrointestinal system MCA, any attempt to wire access beyond the obstruction should be delayed until magnets demonstrate spontaneous fall (Fig. 10).

Impassable colonic obstructions are relatively rare, however can be encountered following surgery and radiotherapy. MCA can be preferred in these patients due to its minimally invasive nature, because of previous surgical procedures or history of radiotherapy that may challenge revision surgery. In colonic obstructions, MCA can be performed with magnets inserted through the anus and an existing enterostomy (Fig. 10).

### Techniques, tips, and tricks during MCA

The most important step in MCA is appropriate patient selection. Long segment obstructions may result in technical failure and procedural complications during magnet removal. The length of stricture is best evaluated when both cranial and caudal wire accesses to the stricture site are obtained. Once the wire accesses are established, magnets are pushed over the wires to the stricture site (supplementary video). Following magnet placement, daily radiographs should be obtained to evaluate magnet apposition. As mentioned above, in MCA of the gastrointestinal system, magnets are expected to demonstrate spontaneous fall following complete adherence, therefore any attempt that could be complicated with perforation should be delayed until spontaneous magnet fall. However, in the setting of biliary and urinary obstructions, an attempt to wire access beyond the obstruction can be performed following complete magnetic adherence on a plain radiograph.

**Fig. 11:**
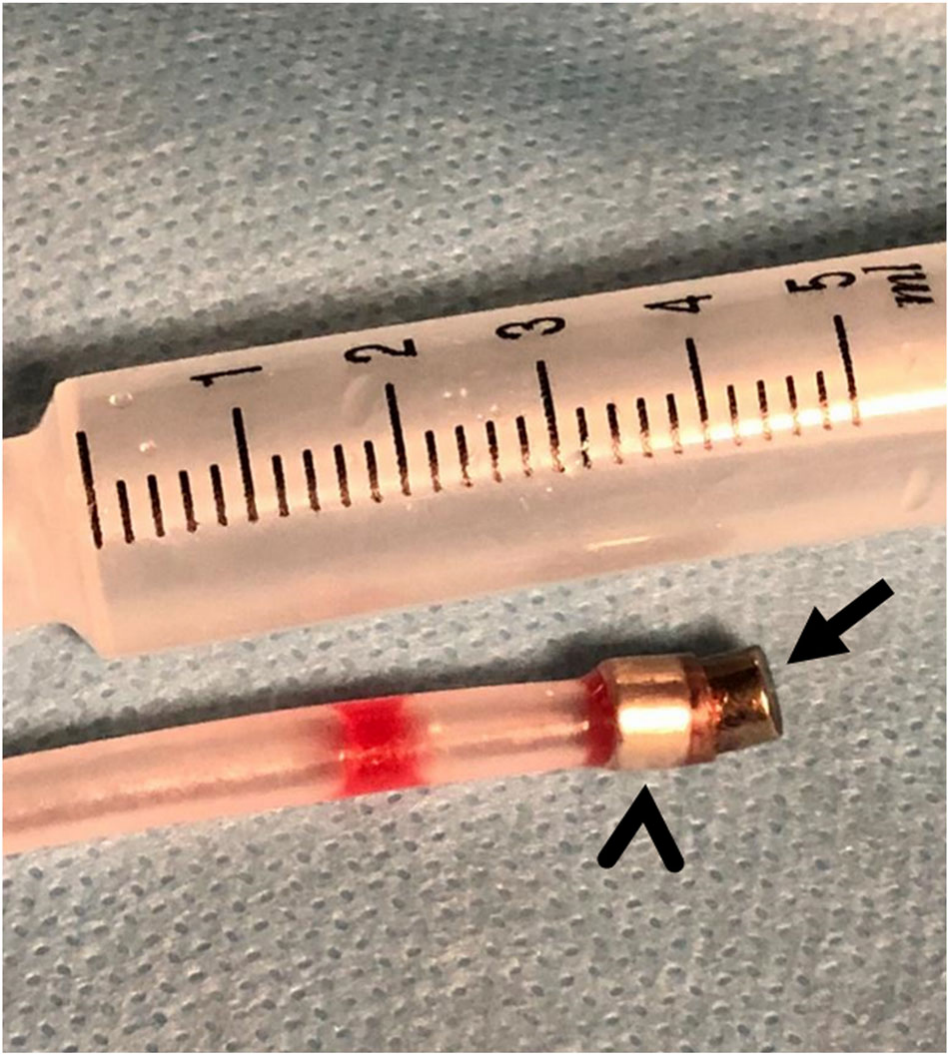
A modified nelaton catheter carrying one fixed 10 F magnet at the tip (arrowhead), is used to grab and remove a dislocated 10 F magnet (arrow)

Cross-sectional images frequently fail to demonstrate the correct distance of the stricture. In case of failure, magnets placed in the distal main bile duct or rectum will probably not raise trouble; however, magnets left within the intrahepatic bile duct, esophagus, or urinary system should be removed in case of failure of magnet adherence. A modified 10 F nelaton catheter carrying one fixed magnet at the tip can be advanced over a guidewire and used to grab and remove magnets under fluoroscopy (Fig. 11).

The magnets placed at the obstruction should be in the correct direction before placement, therefore, before pushing magnets the attraction sites should be determined ex vivo. If magnets face each other with opposite sides, they push each other apart. In this case, a guiding catheter with an angled tip can be used to turn and/or push the magnets once again [2]. Last, in the presence of extravasation and loss of bile duct/ureteral/intestinal integrity, the procedure should be delayed to prevent the magnet fall into the abdominal cavity.

## Conclusion

In this article, various applications of MCA have been reviewed with illustrative cases. Magnets were delivered over wires and the size of magnets ranged from 5 to 18 F. In patients with esophageal, colonic, and ureteroileal anastomosis obstructions, larger-sized magnets can be placed compared to biliary and ureteral obstruction enabling better magnetic adherence and success for longer obstructions.

### Supplementary information

Supplementary videoR1.

## Abbreviations

CBD: Common biliary duct
ERCP: Endoscopic retrograde cholangiopancreatography
MCA: Magnetic compression anastomosis
